# Supplementary material for: Revisiting the invasion paradox: Resistance-richness relationship is driven by augmentation and displacement trends
Source: PLoS Comput Biol. 2024 Jun 12;20(6):e1012193. doi: 10.1371/journal.pcbi.1012193 (PMC11198907; doi:10.1371/journal.pcbi.1012193)
Supplement: S1 Text — Fig A. The chance of four outcomes with respect to Shannon Index under three conditions: the interactions between the resident members a) are more inhibitory (fp = 0.2) b) are equally inhibitory or facilitative (fp = 0.5) c) are more facilitative (fp = 0.8). For each plot 10,000 instances are examined. The Shannon Index of a), b), and c) range between 9.181×10^−4^ and 1.049, between 1.710×10^−4^ and 1.547, and between 1.685×10^−4^ and 1.612, respectively. The Shannon Index range is divided to 30 bins for each condition, and the percentage of each outcome in relation to all instances in each bin range is calculated and plotted. Fig B. The overall trends in invasion outcomes obtained using a Lotka-Volterra (LV) model match those of the mediator-explicit model. In these simulations, similar to Fig 1, a pool of Nsp = 20 species is used as a starting point. The equations used for these simulations were: Ni˙=[ri+1Ki∑jaijNj]Ni, where i and j are the species indices, ri is the species i’s growth rate, Ki is the species i’s carrying capacity, and aij is the interaction coefficient. We assume that aii = −1 and that other interaction coefficients aij are random with a uniform distribution as shown in each panel. The average interaction coefficients is changed from less inhibitory to more inhibitory to examine its impact on invasion outcomes. We simulated the dynamics of this initial pool over 200 generations (20 rounds of growth followed by 1000x dilution) until a stable resident community was reached. The invader was then introduced into the community at a fraction of 0.03% and the outcome was categorized as resistance, disruption, augmentation, or displacement, based on whether the species in the stable community were preserved and whether the invader frequency increased or decreased (as described in Fig 1). Similar to the mediator-explicit model, resident communities with higher richness showed more chance of displacement and less chance of augmentation. This led to an overall no [file pcbi.1012193.s001.pdf]

Revisiting the invasion paradox: resistance-richness relationship is driven by augmentation and displacement trends

Yu Zhu and Babak Momeni

Biology Department, Boston College, Chestnut Hill, MA 02467 United States

Supplementary Information

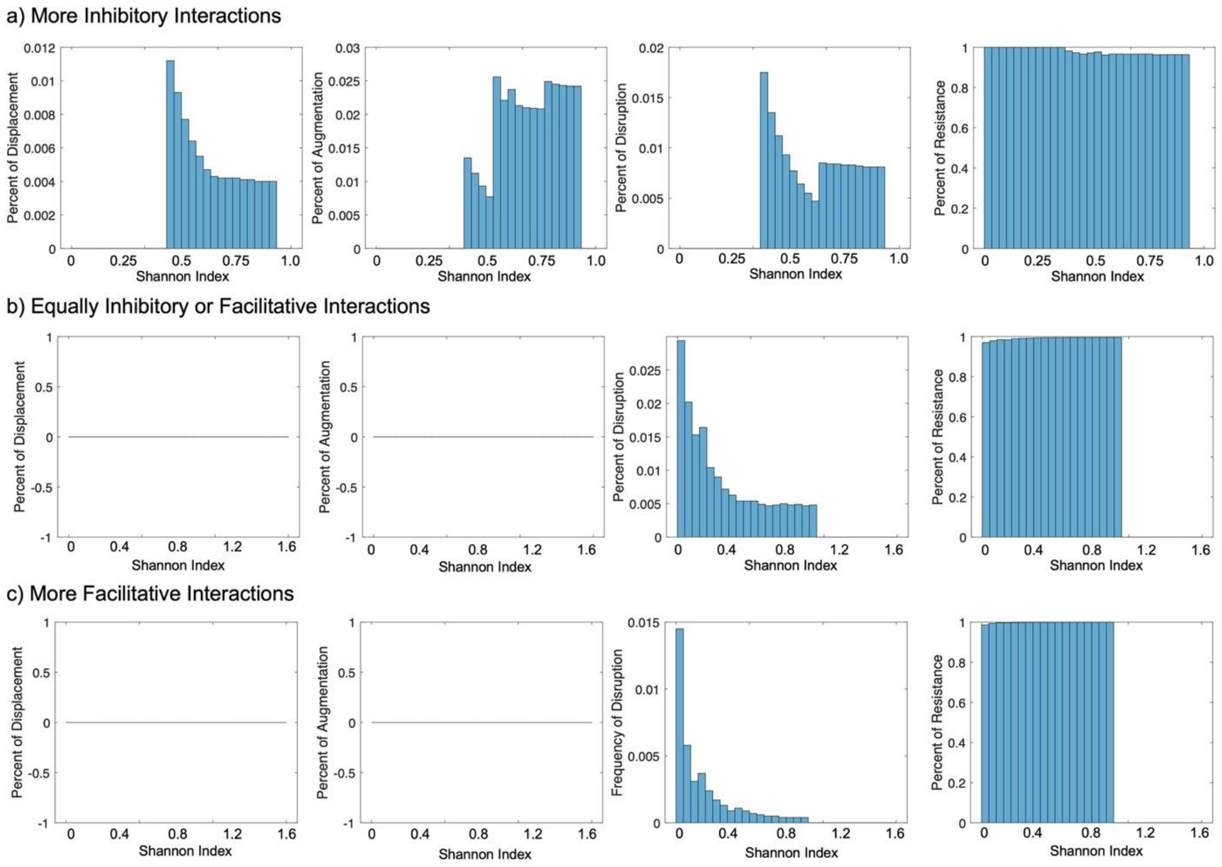

Fig A. The chance of four outcomes with respect to Shannon Index under three conditions: the interactions between the resident members a) are more inhibitory (f_p=0.2) b) are equally inhibitory or facilitative (f_p=0.5) c) are more facilitative (f_p=0.8). For each plot 10,000 instances are examined. The Shannon Index of a), b), and c) range between 9.181×10^{-4} and 1.049, between 1.710×10^{-4} and 1.547, and between 1.685×10^{-4} and 1.612, respectively. The Shannon Index range is divided to 30 bins for each condition, and the percentage of each outcome in relation to all instances in each bin range is calculated and plotted.

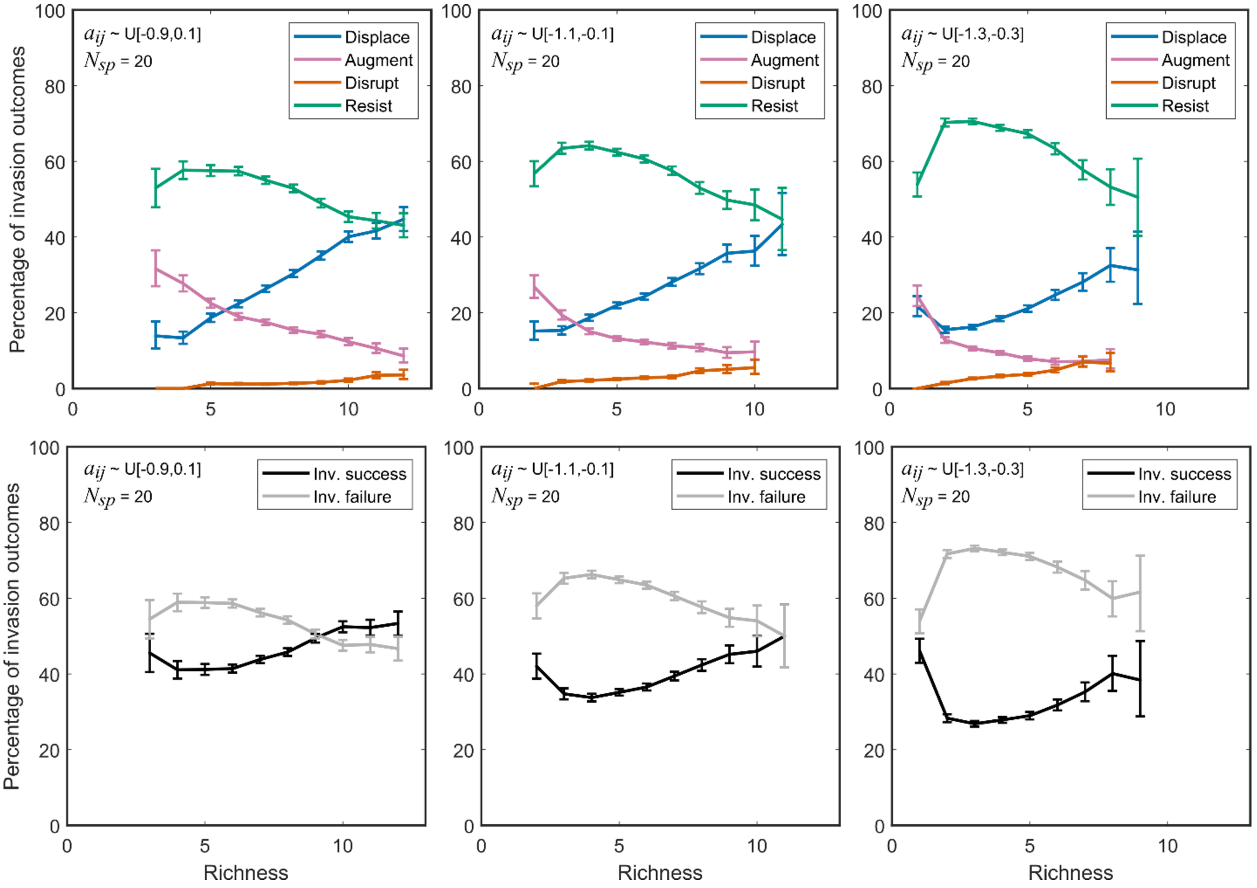

Fig B. The overall trends in invasion outcomes obtained using a Lotka-Volterra (LV) model match those of the mediator-explicit model. In these simulations, similar to Fig 1, a pool of N_{sp} = 20 species is used as a starting point. The equations used for these simulations were: $\dot{N}_i = r_i\left[1 - \frac{N_i}{K_i} + \sum_{j\neq i} a_{ij}N_j\right]N_i$, where i and j are the species indices, r_i is the species i's growth rate, K_i is the species i's carrying capacity, and a_{ij} values are the interaction coefficients. We assume that interaction coefficients a_{ij} are random with a uniform distribution as shown in each panel. The average interaction coefficient is changed from less inhibitory to more inhibitory to examine its impact on invasion outcomes. We simulated the dynamics of this initial pool over 200 generations (20 rounds of growth followed by 1000x dilution) until a stable resident community was reached. The invader was introduced into the community at a fraction of 0.03% and the outcome was categorized as resistance, disruption, augmentation, or displacement, based on whether the species in the stable community were preserved and whether the invader frequency increased or decreased (as described in Fig 1). Similar to the mediator-explicit model, resident communities with higher richness showed more chance of displacement and less chance of augmentation. This led to an overall nonmonotonic resistance-richness relationship which was more pronounced when the interactions within the community were more inhibitory. For each plot, 50,000 instances of invasion are examined. Interactions between resident members and the invaders have the same distribution as the interactions among resident members. The error bars show 95% confidence level estimated assuming a binomial distribution for each outcome. Results are shown only at richness value with at least 30 instances.

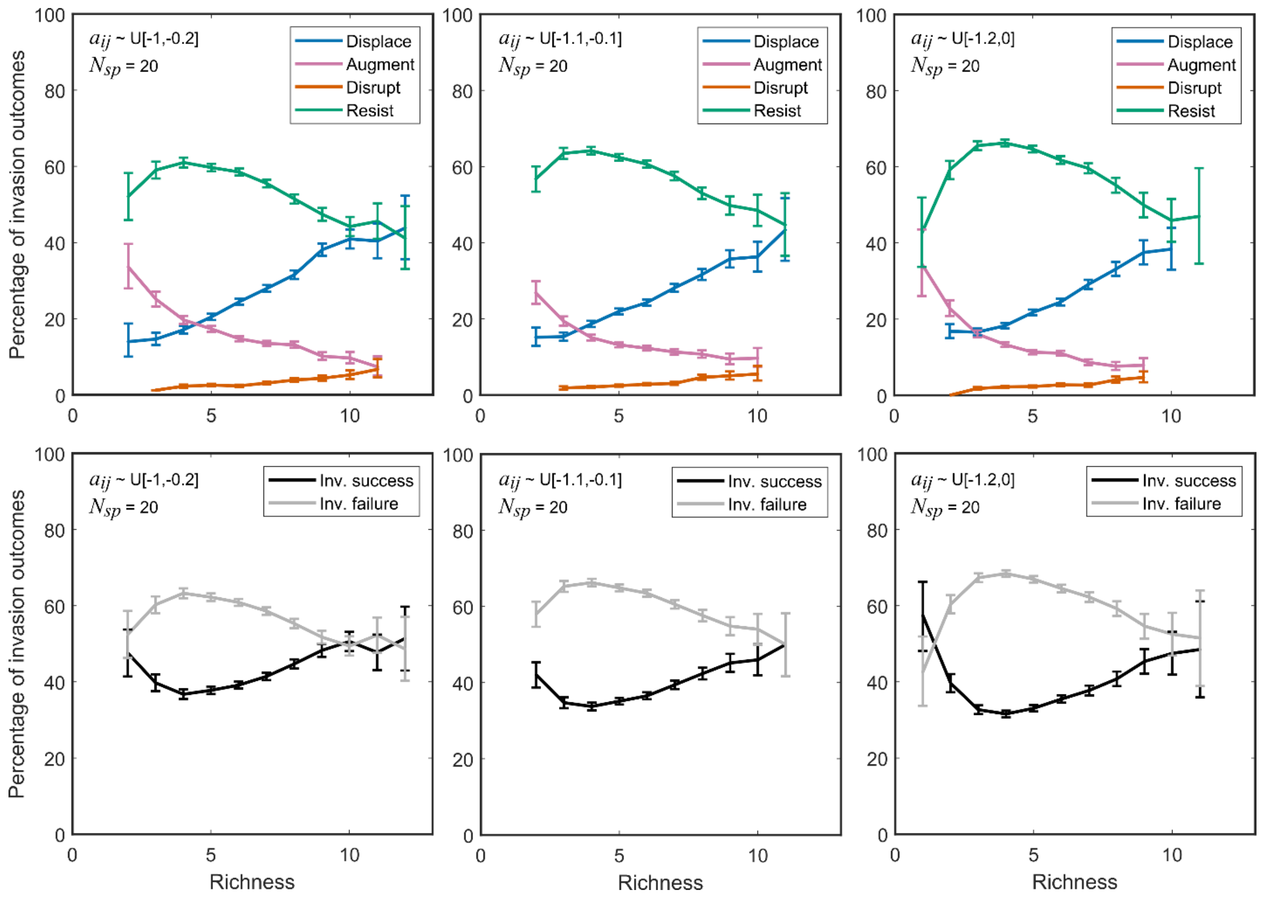

Fig C. The overall trends in invasion outcomes obtained using an LV model match those of the mediator-explicit model. All parameters, equations, and assumptions are similar to Fig B, except that the distribution of off-diagonal interaction coefficients a_{ij} has a uniform distribution with a different spread in each panel. The spread of interaction coefficients is changed from less a narrower range to a wider range to examine its impact on invasion outcomes. Similar to the mediator-explicit model, resident communities with higher richness showed more chance of displacement and less chance of augmentation. This led to an overall nonmonotonic resistance-richness relationship which was more pronounced when the interactions within the community were more inhibitory. For each plot, 50,000 instances of invasion are examined. Interactions between resident members and the invaders have the same distribution as the interactions among resident members. The error bars show 95% confidence level estimated assuming a binomial distribution for each outcome. Results are shown only at richness value with at least 30 instances.

Supplementary Table

Table A. Parameters used for standard simulations

Parameter	Description	Standard Value
N_c	Number of cell types (species) initially includes	20
N_m	Number of mediators	10
N_s	Number of samples	10000
N_g	Number of generations	200
α_m	Average consumption rate of mediators by species (fmol)	0.5
β_m	Average production rate of mediators by species (fmol h^{-1})	0.1
α_{ij}	Consumption rate of chemical *j* by species *i*	$\sim U(0.5\alpha_m, 1.5\alpha_m)$
β_{ij}	Production rate of chemical *j* by species *i*	$\sim U(0.5\beta_m, 1.5\beta_m)$
r_{0m}	Average basal growth rates of cells in the community	0.1
r_{0d}	Deviation of basal growth rates of cells in the community	0.02
r_{0mI}	Average basal growth rates of cells in the invader	0.15
r_{i0}	Population reproduction rate for cells in the community (h^{-1})	$\sim U(r_{0m}-r_{0d}, r_{0m}+r_{0d})$
r_{ij}	Interaction strength of chemical *j* on species *i* (h^{-1})	$\sim U(0,0.2)$ where the fraction of positive sign is determined by f_p
f_p	Probability of positive interactions	0.1
f_{pI}	Probability of positive interactions for the invader	0.5
K_{sat}	Interaction saturation level (fmol h^{-1})	10^4
K_{ij}	Interaction saturation level of chemical *j* by species *i*	$\sim U(0.5K_{sat}, 1.5K_{sat})$
q_p	Probability of production link per population	0.3
q_c	Probability of influence link per population	0.3
q_{pI}	Probability of production link per population for the invader	0.3
q_{cI}	Probability of influence link per population for the invader	0.3
ΣS_{init}	Total initial cell density (ml^{-1})	10^4
ΣS_{dil}	Coculture dilution threshold of cell density (ml^{-1})	10^7
f_{II}	Fraction of the invader when introduced	$3 * 10^{-4}$
