# Supplementary material for: Revisiting the invasion paradox: Resistance-richness relationship is driven by augmentation and displacement trends
Source: PLoS Comput Biol. 2024 Jun 12;20(6):e1012193. doi: 10.1371/journal.pcbi.1012193 (PMC11198907; doi:10.1371/journal.pcbi.1012193)
Supplement: Attachment — Submitted filename: Response to reviewers.pdf [file pcbi.1012193.s002.pdf]

Response to Reviewers' Comments

We thank both reviewers for their constructive suggestions. A point-by-point response to the concerns raised by the reviewers is listed in the following.

[Authors' response is labeled as blue. Modifications to the text are shown as quoted text.]

Reviewer #1:

• Abstract and introduction are well written, easy to follow and motivate the study really well.

Thank you for the positive assessment.

• The phrase "mediator-explicit" model isn't immediately obvious even to me and I am familiar with much of the invasion literature. Suggest defining this term at the first use -- as a model that is mechanism aware?

We added more explanations when we first used that term. In the standard CRM model, the resources only provide benefits to the consumers and the only negative influence between species arises from competition for resources. In contrast, in the mediator-explicit model used in this manuscript each mediator can positively or negatively influence the recipient species. This is meant to capture both positive influences, such as cross-feeding, as well as negative influences, such as inhibitory effect of waste products.

> "…we use a mediator-explicit model [45,46] to track both species and interaction mediators, mediators for short, which are compounds (such as nutrients, metabolic by-products, and toxins) that mediate the interactions between species. Such a model accounts for direct and indirect interactions through mediators in the shared environment. Mediators are produced and consumed by species and can influence species growth positively (as facilitators) or negatively (as inhibitors). We note that the mediator-explicit model is similar to, but distinct from, the standard consumer-resource model (CRM) [47,48], in that in CRM only beneficial resources are included and negative interactions only arise from competition for such resources."
>
> "Mediators are the chemical compounds present in the environment that can interact with species in four possible ways: species either produce or consume mediators and mediators either facilitate or inhibit the growth of species [15,45,46]."

• Line 87-88 -- In service of making the paper more forceful I would strengthen the statements here. The motivation is a bit bigger than just to resolve "some of the ambiguity" about "what invasion success means." I think the authors have already clearly defined what invasion success means, and here we are trying to disambiguate the seemingly contradictory role of richness/diversity. Encourage authors to sharpen this statement if they wish.

Thank you for the suggestion. Clarifying the ambiguity of the relationship between invasion and richness/diversity is our main motivation and we have revised that statement as you suggested.

• Line 92 "structural diversity" - demographic analysis. I'm not sure what this means. Probably my own ignorance, but could you clarify what this is? All the other possible quantifications of diversity make sense to me.

Sorry if we had used a nonconventional terminology. The term “structural diversity” referred to other aspects of population diversity such as age structure and demographic heterogeneity. Since this concept seems to be rarely investigated in invasion ecology and since it does not contribute to the main focus of the paper, we have removed that terminology from the manuscript.

• Lines 108-111. I would just include the explicit model here (Eqn. 1A/B from the Methods). This would be clearer -- and also help address my "mediator-explicit" question above.

Thank you for the suggestion. We have moved the equations to the main text to make the interpretations clearer.

  ○ Again at line 185 -- what does "mediators" mean? I think it means nutrients?

  ○ This ambiguity makes Fig. 4 very hard to understand.

Here, mediator means the chemicals that interact with microbiomes, and they serve as mediators because they implicitly demonstrate the indirect interactions between species. The mediators can interact with microbiomes in four ways, that either are produced or consumed by the species, or they promote or inhibit the growth of species. We did not use the word “nutrients” because the term nutrients is often used for compounds that are needed by (and are beneficial to) the recipient species, but here, mediators can be harmful toxins too.

• Also -- can you clarify in the results section how the simulations were done? Was the community first run to a fixed point (stable coexistence under chemostat or serial dilution) and then the invasion was done? I think simple explanations of this would help the reader.

Thank you for the suggestion. Although the “materials and methods” section has more details, it helps to explain the simulation procedure more clearly in the results section. We have expanded the description of our *in silico* invasion assay in the revised version in Fig 1 and its caption.

> “Top panel shows the overall invasion assay used throughout this manuscript: an initial pool of randomly interacting species is simulated until it reaches stability (200 generations), then an invader is introduced and the invasion outcome is assessed after 200 generations of growth through rounds of growth and dilution.”

  ○ This "+:-" notation is confusing.

  ○ Line 110 -- I don't really know what positive or negative mediator influences mean. Can you explain these in intuitive terms in the context of the model that you used? Competition for shared resources vs. cross feeding of resources seems to be the distinction.

Thank you for bringing up this issue. In our mediator-explicit models (as the equations also illustrate), there are two types of interaction between species and mediators: (1) species produce/consume mediators and (2) mediators facilitate/inhibit the growth of species. The mediator influences here refer to the influence of mediators on species (r_{ij} in Equation 1B). We investigated initial pools of species with different ratio of positive versus negative mediator influences (+:- = 10:90, 50:50, or 90:10) to examine how the invasion trends may change if the interactions among species is shifted from having more facilitation (more "positive" influence of mediators on species) to more inhibition (i.e. more "negative" influence of mediators on species). We have added the following description to make this point clearer in the revised manuscript.

> "We investigated initial pools of species with different ratio of positive versus negative mediator influences (+:- = 10:90, 50:50, or 90:10) to examine how the invasion trends may change if the interactions among species is shifted from having more facilitation (more "positive" influence of mediators on species) to more inhibition (i.e. more "negative" influence of mediators on species)."

And in the caption of Fig 1:

> "In the middle panel, the relationship between richness and different outcomes is examined when the initial pool of resident species has more inhibitory interactions (+:- = 10:90), equal mix of inhibitory and facilitative interactions (+:- = 50:50), and more facilitative interactions (+:- = 90:10)."

• Minor point -- in the Methods authors refer to C_i as "chemicals" but I think more reasonable to call these either nutrients or resources.

You are correct. We had used "chemicals" and "mediators" interchangeably, but that leads to some confusion. In the revised manuscript, we only use "mediators" (short for interaction mediators). To be clear, as mentioned earlier, nutrients or resources are not exactly the same as mediators in our model.

• Fig 1 top -- it would help to make the invader a color that stands out (brighter? Red? Green?) so I can quickly see the instances where invasion succeeds.

Thank you for the suggestion. We have updated Fig 1 top, using the invader as the only dark color to make it easier to see when the invader has succeeded. We have also simplified the figure to make it easier to visually assess changes in the richness of the resident community in the schematic illustration.

• Why does the range of the x-axis vary across the three panels in the bottom of Fig. 1? Is this because for the different interaction profiles it is easier/harder to sustain rich communities?

You are correct. To construct stable resident communities, we start from 20 interacting species in the initial pool, but many of those species go extinct in the process of reaching stability. As a result, the richness of the resulting resident communities will be limited, especially when the initial pool contains many inhibitory interactions. We only retained data points (at each richness value) at which at least 30 stable communities were present

for the analysis to avoid fluctuations caused by small sample sizes. We have now included this information in the legend of our figures.

- ○ Along these lines -- the decline in resistance fraction for richer communities (green lines Fig.1 right two panels bottom) is modest -- it seems worth considering taking these simulations out to higher richness if this is computationally feasible.

Unfortunately, taking the simulations to higher richness comes at a large computational cost. The likelihood of obtaining communities with larger richness drops exponentially with richness. This means that to obtain enough instances to resolve the likelihood of different invasion outcomes at higher richness values, we have to drastically increase the number of initial pools. We have tested the trends for specific instances with more sample sizes, but for the sake of consistency, we have used 10,000 instances for the results reported in this manuscript.

• Paragraph at line 153 is a really nice summary of the main result of this study. Worth emphasizing.

Thank you for the suggestion. We have used this language now in the Abstract and in the Discussion.

• Line 168 -- there is not Table 1 that I can find.

Sorry about that. The table was accidentally dropped in the assembly of the final file. We have added the table back as Fig S1.

• The setup to fig. 1 is confusing. The authors measure stability in TWO ways -- reducing the proportion of randomly species OR invading. The fact that the authors including invasion in a measure of stability and then measured invasion resistance for "stable" and "unstable" communities seems tautological. Haven't they defined stability as invasion resistance and then claimed that stable communities are invasion resistant? If the stability assessment of communities only included perturbations to abundances of members of the community there would be no problem here!

Sorry about the confusion. To make our assay less sensitive to the initial conditions, we start from a pool of species, and follow the initial assembly for 200 generations to reach a stable resident community. We then introduce the invader and examine the state of both the resident community and the invader, as outlined in Fig 1 (top panel). We have updated Fig 1 in the revised version to explicitly show the first stage as well.

In Figure 2, two separate aspects are investigated: (1) whether the community is maintained after introducing a perturbation (of magnitude 0.1 in terms of Bray-Curtis dissimilarity) and (2) whether the community is maintained after introducing an invader into it. Our findings from Fig 2 show that communities that are sensitive to perturbations in their composition are also more prone to invasion. In contrast, those robust to changes in population composition also appear to be able to resist invaders more effectively. To avoid confusion between Fig 1 and Fig 2, we have changed the designation in Fig 2 to ‘robust’ versus ‘not robust’ instead of ‘stable’ versus ‘unstable’.

- In Figs. 1, 3,4. -- If you chose line colors that were similar for cases where the invader succeeded vs failed this would really help in reading the plots. Alternatively, you could include panels that showed the classic success rate vs richness (irrespective of mechanism) below each panel. This might be nice. Up to the authors.
    - E.g. in line 262 authors are claiming a trend that requires the reader to "add up" two lines in the plots. This is a bit confusing and weakens the results.

Thank you for the suggestion. We have included additional panels in Fig 1, showing the total fractions of invasion success versus failure. Or the sake of space, we have not repeated these panels for Figures 3 and 4, but we hope that the main message is clear enough so that repeating it is not necessary.

- The result in Fig. 5 is nice -- is interesting that increasing the positive interactions with the invaders increases the displacement fraction -- so faciliatory interactions with the invader increase the rate at which members of the community are displaced. Essentially members of the community are helping the invader displace other members of the community. Just a comment.

Thank you for the comment. We have added this point to Discussions to highlight that, perhaps contrary to intuition, more facilitation of invader does not necessarily lead to augmentation and the more common outcome in such a case is displacement.

> "...when the invader benefits from facilitation by the resident species, augmentation and displacement become more prevalent, as the invader is assisted by the community itself to establish (Fig 5). Among these cases, in communities with higher richness displacement can become the dominant outcome."

- Line 280 -- when the invader introduces a new metabolite -- I don't understand under what conditions a new metabolite is introduced by the invader. I think this has to do with my confusion about what a mediator is.

In our standard simulations, there are a fixed number of mediators in the simulation. Each of these mediators are produced by a subset of species present in that simulations instance (stochastically assigned) and influence a subset of species either positively (facilitation) or negatively (inhibition). In our standard simulations, the invader is treated similar to any of the resident members; i.e. it produces a subset of the existing mediators in the simulation and gets influenced by a subset of the existing mediators.

In simulations with a new mediator, we added a new mediator into the simulation when the invader was introduced. This mediator is produced by the invader, and randomly influences a subset of the resident species. Loosely, this new mediator represents a new niche created by the invader; we examined its influence on invasion outcomes.

- Line 303 -- the model used here looks like a variant of a CRM to me, so this statement is confusing.

Sorry about the confusion. In the standard CRM model, the resources only provide benefits to the consumers and the only negative influence between species arises from competition for resources. In contrast, the mediator-explicit model is used in this manuscript, which was introduced in (Momeni et al., eLife, 2017) and expanded with experimental support for model assumptions in (Niehaus et al., Nature Communications, 2019). In the mediator-explicit model, unlike CRM, there is no strict hierarchy for the mediators, but each mediator can positively or negatively influence the recipients. This is meant to capture both positive influences, such as cross-feeding, as well as negative influences, such as inhibitory effect of waste products.

We have added the following lines and moved the description of the model to the main text in the revised manuscript to make this point clear:

> "We simulate the *in silico* microbial communities using the mediator-explicit model (Equation 1). Mediators are the chemicals present in the environment that can interact with species in four possible ways, that species either produce or uptake mediators and mediators either promote or inhibit the growth of species [15,45,46]. Specifically, the rate of change of species i's concentration is proportional to the cell densities of the species (S_i) scaled by its basal growth rate (r_{i0}) in addition to the sum of mediator j's influence, which is the product of interaction strength on species i (r_{ij}) and the concentration of mediators (M_j) controlled by the saturation concentration (K_{ij}) (Equation 1B). Similarly, the rate of change of mediators i is total results of individual species j if they produce the mediator i with a rate of β_{ij} or consume it with a rate of α_{ij} (Equation 1A).
>
> $$\frac{dM_i}{dt} = \sum_j \left[\beta_{ij} - \alpha_{ij}\frac{M_i}{M_i+K_{ij}}\right] S_j \qquad (1A)$$
>
> $$\frac{dS_i}{dt} = \left[r_{i0} + \sum_j r_{ij}\frac{M_j}{M_j+K_{ij}}\right] S_i \qquad (1B)$$"

And

> "Using the mediator-explicit model, we first generated stable resident communities with multiple species *in silico*. For this, we assembled interacting species with random interactions and simulated the dynamics until a subset of species reached stable coexistence. In these simulations, we investigated initial pools of species with different ratio of positive versus negative mediator influences (+:- = 10:90, 50:50, or 90:10) to examine how the invasion trends may change if the interactions among species is shifted from having more facilitation (more "positive" influence of mediators on species) to more inhibition (i.e. more "negative" influence of mediators on species). We then introduced the invader into these resident communities and simulated the dynamics to assess the invasion outcomes (Fig 1, top)."

As a side-note, our lab is investigating invasion using the standard CRM model in a separate manuscript. We have observed similar patterns, but there are additional considerations based on the structure of the resources and the architecture of species dependencies which required more in-depth investigations beyond the scope of this manuscript.

• An aside -- it appears this work was done largely by an undergraduate. Impressive for someone early in their training. Well done!

Thank you for your encouragement.

Reviewer #2

Zhu and Momeni, using a consumer-resource model, explore how diversity and the type of interactions affect the outcome of invasions. Invasion outcomes are not limited to the successful or unsuccessful establishment of the new species, but include also what happens to the resident community, for example whether native species go extinct or not when the invader is successful. The main claim is that, as diversity of the native community increases, augmentation (that is when native species survive the invasion) becomes less likely, while displacement (that is when some native species do not survive the invasion) becomes more likely. Surprisingly, the probability of resistance is not affected by species richness, but it strongly influenced by the fraction of positive interactions.

The paper is well written, but the narrative could be improved. The various simulations could be better connected and figures would benefit from a clear schematic of what is the simulation doing in any particular instance. Some interesting results are presented, but the model the authors used was developed in a previous paper and some of the trends that are discovered are weak.

Thank you for the suggestions. To make the overall layout of the invasion assay clearer, we have added it to Fig 1. All simulations in this manuscript use the same overall assay.

I have three suggestions:
1. Explain clearly what has been done in the simulations described in "Communities that are more stable are also more resistant to invasion". From the current version of the text, it is unclear whether after the two possible perturbations (reduction of the biomass of one species and invasion) there is a second invasion.

In response to your comment and the comment from Reviewer #1, we have moved the description of the model and the invasion assay to the main text and updated Figure 1 to show the overall assay used for our investigations:

> "We simulate the *in silico* microbial communities using the mediator-explicit model (Equation 1). Mediators are the chemicals present in the environment that can interact with species in four possible ways, that species either produce or uptake mediators and mediators either promote or inhibit the growth of species [15,45,46]. Specifically, the rate of change of species i's concentration is proportional to the cell densities of the species (S_i) scaled by its basal growth rate (r_{i0}) in addition to the sum of mediator j's influence, which is the product of interaction strength on species i (r_{ij}) and the concentration of mediators (M_j) controlled by the saturation concentration (K_{ij}) (Equation 1B). Similarly, the rate of change of mediators i is total results of individual species j if they produce the mediator i with a rate of β_{ij} or consume it with a rate of α_{ij} (Equation 1A).

$$\frac{dM_i}{dt} = \sum_j \left[\beta_{ij} - \alpha_{ij} \frac{M_i}{M_i+K_{ij}}\right] S_j \qquad (1A)$$

$$\frac{dS_i}{dt} = \left[r_{i0} + \sum_j \ r_{ij} \frac{M_j}{M_j + K_{ij}} \right] S_i \qquad (1B)”$$

And

> “Using the mediator-explicit model, we first generated stable resident communities with multiple species *in silico*. For this, we assembled interacting species with random interactions and simulated the dynamics until a subset of species reached stable coexistence. In these simulations, we investigated initial pools of species with different ratio of positive versus negative mediator influences (+:- = 10:90, 50:50, or 90:10) to examine how the invasion trends may change if the interactions among species is shifted from having more facilitation (more “positive” influence of mediators on species) to more inhibition (i.e. more “negative” influence of mediators on species). We then introduced the invader into these resident communities and simulated the dynamics to assess the invasion outcomes (Fig 1, top).”

We have also updated Fig 2 to clarify that one of the measures is robustness to perturbation (now labeled as ‘robust’ in the figure to avoid confusion with the term ‘stable’ used for initially establishing the resident communities) and the other measure is resistance to invaders. There is no second invasion involved in our investigation.

2. Explain how the outcomes presented in this paper would change when “mediators” are not explicit, like in the case of the Lotka Volterra model

In response to your comment and the comment from Reviewer #1, we have added two supplementary figures (Fig S2 and Fig S3) in which the overall assay in Fig 1 is simulated using a Lotka-Volterra model. Overall, the main findings in the mediator-explicit model (i.e. less augmentation and more displacement for resident communities with higher richness) is consistent across these models. Our preliminary investigation showed that our findings still hold upon changing the average interspecies interaction coefficients or changing the spread of interspecies interaction coefficients in the Lotka-Volterra model.

3. I find the word “mediator” instead of resources quite misleading. Why are the authors using this term instead of “resource”?

The terminology is intended to specify all compounds that mediate species interactions. This is in contrast to resources that are considered to be strictly beneficial to the recipients. We have added the following to the manuscript to make this distinction clear:

> “…we use a mediator-explicit model [45,46] to track both species and interaction mediators, mediators for short, which are compounds (such as nutrients, metabolic by-products, and toxins) that mediate the interactions between species. Such a model accounts for direct and indirect interactions through mediators in the shared environment. Mediators are produced and consumed by species and can influence species growth positively (as facilitators) or negatively (as inhibitors). We note that

the mediator-explicit model is similar to, but distinct from, the standard consumer-resource model (CRM) [47,48], in that in CRM only beneficial resources are included and negative interactions only arise from competition for such resources.”
